# Is biochemical hypoglycemia necessary during an insulin tolerance test?

**DOI:** 10.20945/2359-3997000000200

**Published:** 2020-03-04

**Authors:** Yasin Simsek, Zuleyha Karaca, Halit Diri, Fatih Tanriverdi, Kursad Unluhizarci, Fahrettin Kelestemur

**Affiliations:** 1 Erciyes University Medical School Department of Endocrinology Kayseri Turkey Erciyes University Medical School, Department of Endocrinology, Kayseri, Turkey

**Keywords:** Insulin tolerance test, hypoglycemia, hypopituitarism, dynamic test

## Abstract

**Objective:**

The insulin tolerance test (ITT) has been accepted as the gold standard test for assessing the integrity of the growth hormone (GH) – insulin-like growth factor (IGF-1) axis and the hypothalamic-pituitary-adrenal (HPA) axis. The goal of the test is to achieve clinical and biochemical hypoglycemia at a blood glucose level ≤ 40 mg/dL to effectively and correctly assess the HPA and GH-IGF-1 axes. In this study, the GH and cortisol responses of patients who achieved and failed to achieve biochemical hypoglycemia during an ITT were compared.

**Subjects and methods:**

One hundred thirty-five patients with pituitary disorders were included in the study. Samples for blood glucose levels were obtained after clear symptoms of clinical hypoglycemia developed. The patients were enrolled in the hypoglycemic and nonhypoglycemic groups according to whether their plasma glucose level ≤ 40 mg/dL or > 40 mg/dL during an ITT, and the groups were compared in terms of their GH and cortisol responses.

**Results:**

The mean age, body mass index and waist circumference of the two patient groups were found to be similar. The mean blood glucose level was significantly lower in the hypoglycemic group than in the nonhypoglycemic group (19.3 and 52.0 mg/dL, respectively). When the two groups were compared in terms of peak cortisol and GH responses, no statistically significant differences were found.

**Conclusion:**

The data presented suggest that clinically symptomatic hypoglycemia is as effective as biochemically confirmed hypoglycemia during an ITT. Arch Endocrinol Metab. 2020;64(1):82-8

## INTRODUCTION

The insulin tolerance test (ITT), glucagon stimulation test (GST), adrenocorticotropic hormone (ACTH) stimulation test, and metyrapone test are used primarily to assess the functional integrity of the HPA axis ( 1 , 2 ). The ITT, which was developed in 1960 ( 3 ), is performed by intravenous insulin administration leading to a decline in blood glucose levels. Approximately 20 to 60 minutes after insulin administration, low blood glucose triggers the secretion of glucagon, epinephrine, norepinephrine, cortisol, GH, and ACTH in normal subjects ( 4 ). As hormonal responses to hypoglycemia, GH and cortisol levels can be measured by an ITT.

According to recent guidelines, severe GH deficiency in adults is defined as a peak GH response less than 3 µg/L on an ITT, while healthy subjects have been shown to respond with a peak GH exceeding 5 µg/L ( 5 - 7 ). Various cut-off values for the peak cortisol levels have been suggested for ITTs, and they range between 15 and 23 µg/dL for a normal response to an ITT in healthy subjects ( 8 ). Currently, the ITT is considered the gold standard test for diagnosing GH and cortisol deficiencies ( 3 , 8 - 10 ).

An ITT is unpleasant for patients and is not free from side effects; therefore, close medical supervision by experienced medical staff is required, and the test is contraindicated in elderly patients and in patients with cerebrovascular disease, epilepsy or ischemic heart disease ( 11 ). In general, the goal is to achieve a blood glucose level ≤ 2.2 mmol/l (40 mg/dL) during an ITT for an effective assessment of the HPA and GH-IGF-1 axes ( 12 , 13 ). Although glucose levels of 2.2 mmol/l (40 mg/dL), 2.5 mmol/l (45 mg/dL), and 2.8 mmol/l (50.4 mg/dL) were suggested for effective hypoglycemia during an ITT, a 2.2 mmol/l level has been widely used as the threshold level for the biochemical criterion worldwide ( 1 , 14 ). If adequate biochemical hypoglycemia (plasma glucose, ≤ 2.2 mmol/l) is not achieved, it would be necessary to give a second injection of insulin ( 15 ). Further insulin administration may cause severe hypoglycemia, and an intravenous dextrose infusion may be necessary during the test, before finishing. Although dextrose infusion has been reported to have no effect on the glucose level, it would make the test results more complicated. In this study, we aimed to show whether hypoglycemia-related manifestations are suitable or biochemical hypoglycemia is required to achieve accurate peak cortisol and GH responses during an ITT.

## SUBJECTS AND METHODS

Retrospective analyses of 135 patients (83 female, 52 male) in whom an ITT was performed for suspected hypopituitarism were included in the study. Approval from the local Ethics Committee was obtained before conducting the study. All patients developed clear symptoms of hypoglycemia, including tachycardia, anxiety, sweating, faintness, weakness, dizziness, nausea, hunger, drowsiness, confusion, headache, and impaired speech and vision during the ITT, and blood glucose levels were analyzed during these symptoms. According whether plasma glucose levels lower or higher than 40 mg/dL were achieved during the test, two groups were created: the first group had glucose levels lower than 40 mg/dL (n: 118), and the second group had > 40 mg/dL (n: 17). Group 2 (BG > 40 mg/dL) consisted of only 17 patients, compared to 118 in group A, making the calculation of the statistical significance of the difference in hormone responses questionable.

The ITT was performed after an overnight fast, and blood samples for serum cortisol and GH measurements were obtained prior to the intravenous administration of 0.1 U/kg (0.2 U/kg if BMI > 30 kg/m^2^) soluble regular insulin (minute -15), as well as immediately after symptomatic hypoglycemia began (minute 0), and after 30, 60, 90 and 120 minutes. An additional dose of insulin was given to patients who had neither sufficient biochemical hypoglycemia (≤ 40 mg/dL) nor hypoglycemia symptoms but not to patients who developed hypoglycemia symptoms. Symptomatic hypoglycemia was determined by clinical findings (especially palpitation, sweating, tachycardia, anxiety), whereas biochemical hypoglycemia was determined by the plasma glucose measurement. A serum cortisol level ≥ 18.0 µg/dL and a serum GH level ≥ 3.0 µg/L were accepted as sufficient responses to the ITT ( 1 , 13 ).

The assay method and commercial kit used for the serum GH were the immunoradiometric assay (IRMA) and Immunotech SAS (Marseille, France), respectively, and the values of the intra-assay and interassay coefficients of variations were 1.5% and 14%, respectively. The method of assay, commercial kit, and intraassay and interassay coefficients of variations for IGF-1 were as follows: IRMA, Immunotech SAS (Marseille, France), and 6.3% and 6.8%, respectively. For the serum cortisol level, the assay method, commercial kit, and intraassay and interassay coefficients of variations were radioimmunoassay, Immunotech sro (Czech Republic), and 5.8% and 9.2%, respectively.

### Statistical analysis

Descriptive statistics included the mean, standard deviation or error, frequency, and percentage. Mann–Whitney U tests were used for comparisons between the groups for quantitative variables. Chi-squared and Fisher’s test were used for the analysis of ratios. The area under the curve (AUC) was calculated according to the trapezoid formula. All statistical analyses were performed with SPSS 20.0 software (Chicago, USA). All analyses were performed within a 95% confidence interval. A p value of < 0.05 was considered statistically significant.

## RESULTS

The demographics of the patients were similar when the 2 groups were compared ( Table 1 ). Both groups also had similar ratios in terms of pituitary disorders, including tumoral or nontumoral, and operational status. In group 1, 85 of the patients had tumoral causes (27 nonfunctioning adenoma, 37 prolactinoma, 16 acromegaly and 5 Rathke’s cleft cyst), and 33 of the patients had nontumoral causes (10 empty sella, 9 congenital pituitary failure, 6 Sheehan’s syndrome, 2 traumatic brain injury, 3 idiopathic hypogonadotropic hypogonadism and 3 lymphocytic hypophysitis). In group 2, 12 patients had tumoral causes (8 nonfunctioning adenoma, 2 prolactinoma, 1 acromegaly and 1 Rathke’s cleft cyst), and 5 patients had nontumoral causes (1 empty sella, 2 Sheehan’s syndrome, 1 traumatic brain injury and 1 idiopathic hypogonadotropic hypogonadism). All patients with pituitary tumors were in remission due to medical or surgical treatment. The two groups were similar in terms of the frequencies of hypopituitarism. The mean blood glucose level of the hypoglycemic group (19.3 ± 0.9 mg/dl) was significantly lower than that of the nonhypoglycemic group (52.0 ± 2.3 mg/dL). The basal hormone levels of the patients were similar in both groups ( Table 2 ). There was no statistically significant difference in terms of peak GH levels during the ITT between the 2 groups, even considering acromegaly ( Tables 3 and 4 ). In addition, when the two groups were compared in terms of peak cortisol, no statistically significant differences were found ( Table 5 ).

**Table 1:** Demographic data of the groups

	Group 1 n: 118	Group 2 n: 17	p value
Male	45 (38.1%)	7 (41.2%)	0.81
Female	73 (61.9%)	10 (58.8%)	0.81
Age (years)	42.6 ± 10.8	43.5 ± 12.4	0.73
Height (cm)	162.6 ± 8.8	164.9 ± 9.8	0.36
Weight (kg)	79.5 ± 15.6	78.6 ± 18.9	0.84
BMI (m^2^/kg)	30.0 ± 6.1	28.7 ± 6.6	0.47
Waist circumference (cm)	95.7 ± 12.7	97.8 ± 13.9	0.55

BMI: body mass index.

**Table 2:** Mean serum glucose (at hypoglycemia) and basal hormone levels of the patients

	Group 1 n: 118	Group 2 n: 17	p value
TSH (0.57–5.6 lU/mL)	1.5 ± 1.1	1.1 ± 1.2	0.22
fT4 (0.88–1.72 ng/dL)	1.1 ± 0.2	1.1 ± 0.1	0.96
IGF-I (64–336 ng/mL)	151.7 ± 105.3	133.3 ± 100.2	0.33
Cortisol (5–25 µg/dL)	10.0 ± 6.1	9.8 ± 5.6	0.59
Blood glucose (mg/dL)	19.3 ± 0.9	52.0 ± 2.3	< 0.001

TSH: thyroid-stimulating hormone; ft4: free thyroxine; IGF-1: insulin-like growth factor-1.

**Table 3:** GH responses (µg/L) for the patients without acromegaly only

Minutes	-15	0	30	60	90	120	Peak GH	AUC
Group 1 n: 102	0.2 ± 0.9	0.6 ± 0.3	0.9 ± 0.2	0.7 ± 0.2	0.4 ± 0.2	0.2 ± 0.0	1.3 ± 0.2	96.4 ± 26.0
Group 2 n: 16	0.9 ± 0.5	1.7 ± 0.1	1.4 ± 0.5	0.5 ± 0.2	0.4 ± 0.2	0.1 ± 0.0	1.6 ± 0.5	142.8 ± 60.5
P value	0.12	0.49	0.09	0.33	0.31	0.97	0.56	0.26

Data were expressed as the mean ± SEM; AUC: area under the curve.

**Table 4:** GH responses (µg/L) of all patients (with or without acromegaly)

Minutes	-15	0	30	60	90	120	Peak GH	AUC
Group 1 n: 118	0.3 ± 0.0	0.6 ± 0.3	0.9 ± 0.2	0.7 ± 0.2	0.5 ± 0.1	0.3 ± 0.0	1.3 ± 0.2	98.3 ± 23.0
Group 2 n: 17	0.9 ± 0.5	1.6 ± 1.0	1.3 ± 0.4	0.5 ± 0.2	0.4 ± 0.1	0.1 ± 0.0	1.5 ± 0.5	137.1 ± 56.3
P value	0.14	0.50	0.11	0.33	0.43	0.88	0.62	0.32

Data were expressed as the mean ± SEM; AUC: area under the curve.

**Table 5:** Cortisol responses (µg/dL) of the patients during an ITT

Minutes	-15	0	30	60	90	120	Peak F	AUC
Group 1 n: 118	10.1 ± 0.5	10.1 ± 0.6	10.7 ± 0.6	8.8 ± 0.6	7.8 ± 0.4	7.0 ± 0.4	12.1 ± 0.7	1394.4 ± 78.5
Group 2 n: 17	10.0 ± 1.5	11.6 ± 2.0	14.7 ± 2.0	14.0 ± 2.0	12.0 ± 1.9	10.1 ± 1.4	16.4 ± 2.2	1897.1 ± 266.8
P value	0.97	0.40	0.46	0.02	0.04	0.04	0.05	0.03

Data were expressed as the mean ± SEM; F: cortisol; AUC: area under the curve.

In group 1, peak GH levels were achieved at the 0th (basal) minute in 14.7%, at the 30th minute in 45.5%, at the 60th minute in 15.6%, at the 90th minute in 9.5%, and at the 120th minute in 14.7% of the patients, and in group 2, peak GH levels were achieved at the 0th (basal) minute in 11.8%, at the 30th minute in 41.1%, at the 60th minute in 41.1%, and at the 120th minute in 5.9% of the patients. Peak cortisol levels were achieved in group 1 at the 0th minute in 42.4%, at the 30th minute in 35.9%, at the 60th minute in 6.1%, at the 90th minute in 7.8%, and at the 120th minute in 7.8% of the patients, and in group 2, peak cortisol levels were achieved at the 0th minute in 11.7%, at the 30th minute 41.1%, at the 60th minute 41.1%, and at the 120th minute 5.8% of the patients. No correlation was detected among blood glucose levels after insulin and at peak cortisol and GH levels (p > 0.05).

In group 1, peak GH levels were mostly measured at the 30th minute, and peak cortisol levels were measured at the 0th minute; in group 2, peak GH levels were measured at the 30th-60th minute, and cortisol levels were measured at the 30th-60th minute of the ITT. According to these results, group 1 achieved peak GH and cortisol levels earlier than group 2.

**Figure 1:**
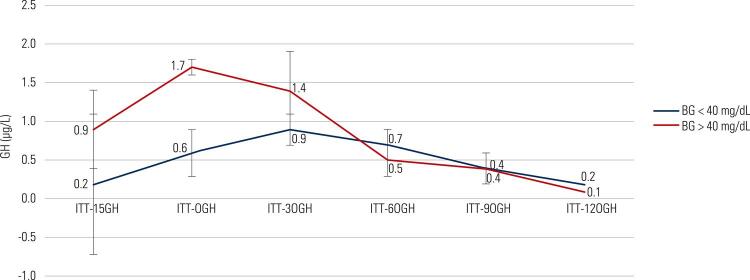
GH responses in patients without acromegaly.

BG: blood glucose; GH: growth hormone.

**Figure 2 f02:**
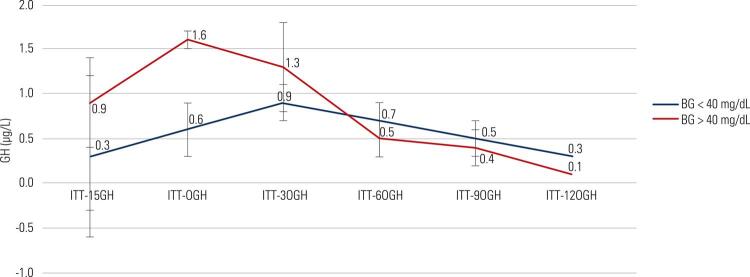
GH responses in patients with acromegaly.

BG: blood glucose; GH: growth hormone.

**Figure 3:**
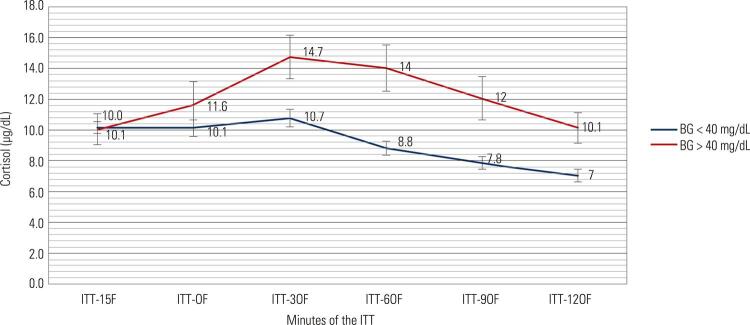
Mean cortisol responses in ITT.

BG: blood glucose; F: cortisol.

The mean cortisol responses of every test minute, peak cortisol levels and AUC values were significantly higher in group 2 than in group 1 ( Figure 3 ). Peak GH responses were similar in both groups regardless of acromegaly; however, group 2 had higher AUC levels of GH than group 1 (p < 0.05) ( Tables 3 and 4 ) ( Figures 1 to 3 [Fig f02] ).

When comparing the patients with inadequate responses to GH and cortisol levels (i.e., peak GH response less than 3 µg/L and peak cortisol levels less than 18.0 µg/dL), after removing patients with adequate responses in the tests, there was no statistically significant difference between groups in terms of GH and cortisol levels (p: 0.56, p: 0.84, respectively).

## DISCUSSION

Among various provocative tests of GH and cortisol secretions, the ITT is considered the gold standard. It can evaluate the whole hypothalamus-pituitary-end organ axis ( 16 ). Insulin injections are intended to induce excessive hypoglycemia, which leads to a major stress response of the body, with increases in ACTH, cortisol and GH levels, and activation of the sympathetic nervous system ( 17 ). Most previous studies reported that blood glucose should decrease to 2.2 mmol/L (40 mg/dL) to activate a stress response, which might be essential at the beginning of an ITT ( 3 , 7 , 12 , 13 ). In our endocrinology department, we also use this cut-off value for ITTs, and most of the patients in the present study (84.6%) developed blood glucose levels < 2.2 mmol/L during the ITT.

Under certain conditions, there may be discordance between blood glucose levels and hypoglycemia symptoms. Hypoglycemia is usually described as a plasma glucose level < 70 mg/dL (3.9 mmol/L) ( 18 ). As blood glucose levels decrease, the activation of the autonomic nervous system leads to neurogenic symptoms such as palpitations, sweating, hunger, and anxiety, which allows the perception of hypoglycemia and the reversal of the symptoms after restoration of the blood glucose level to normal ( 19 ). On the other hand, hypoglycemia unawareness is described as the onset of neuroglycopenia before the appearance of autonomic warning symptoms with a significant decrease in blood glucose ( 20 ). In patients with diabetes mellitus, recurrent hypoglycemia has been shown to reduce the glucose level that precipitates the counter regulatory response necessary to restore euglycemia during subsequent episodes of hypoglycemia ( 21 , 22 ).

Several studies revealed that insulin has direct effects on pituitary functions ( 23 , 24 ). Schultes and cols. showed that intravenous infusion of high doses of insulin to healthy persons, even in the presence of euglycemia, increases plasma ACTH and cortisol concentrations. Additionally, they demonstrated that differences in cortisol levels between the high and the low doses of the insulin infusion could not be explained by differences in ACTH levels; insulin-induced cortisol release may be related to a direct influence on the adrenals ( 25 ). The strong stimulatory effect of hypoglycemia might have covered the moderate stimulatory effect of insulin on HPA secretory activity ( 25 ). An experimental study suggested that insulin also increases the HPA secretory response to moderate hypoglycemic stress ( 26 ). Another study demonstrated the prolonged stimulatory effect of insulin on HPA secretory activity, preventing the development of hypoglycemia-associated counter regulatory failure ( 27 ). Insulin crosses the blood-brain barrier, and its receptors were found in the hippocampus, hypothalamus, and pituitary ( 28 , 29 ). Therefore, insulin seems to affect pituitary functions directly ( 30 ). Eventually, the cause of similar cortisol responses at different glucose levels may be related to individual differences in the distribution of insulin receptors in the brain and the direct effect of insulin on the adrenal glands.

The counterregulatory response to hypoglycemia starts when the serum glucose level falls below 63 mg/dL (3.5 mmol/l). Hormonal responses to hypoglycemia are given step by step. The first response to falling blood glucose levels is decreasing insulin levels. When the glucose level continues to fall, glucagon is released, and catecholamines increase ( 31 ). Despite the rapid effects of glucagon and catecholamines on glucose regulation, the effects of cortisol and growth hormone during hypoglycemia are delayed ( 30 , 32 ). The magnitude of this response depends on the depth of hypoglycemia achieved ( 32 , 33 ). However, Amiel and cols. revealed that the response of cortisol and GH was not dependent on the extent of the drop in glucose levels ( 33 ). In this study, despite having lower glucose levels, the group 1 patients had lower peak and AUC values for GH and cortisol levels than the other groups. According to this study, the magnitude of the response of the counter regulatory system to hypoglycemia was not dependent on the depth of hypoglycemia. In a study by Lee and cols., fasting blood glucose was found to be the most important determinant of the dose of insulin required to achieve adequate biochemical hypoglycemia (blood glucose < 2.2 mmol/l) during an ITT ( 34 ). The ITT inevitably leads to uncontrolled hypoglycemia, which is potentially dangerous, and it is an unpleasant test for both the patients and medical staff. Ajala and cols. also found that the hypoglycemia achieved during an ITT was much lower than the target required ( 2 ). Borm and cols. reported that the low dose infusion of glucose did not change peak cortisol and GH responses during an ITT; however, their study included only 16 healthy subjects, and no patients with hypopituitarism were involved ( 35 ).

In conclusion, the present data suggest that clinically symptomatic hypoglycemia may be used in place of biochemically confirmed glycemic levels ≤ 40 mg/dL during an ITT. Therefore, further insulin administration may not be recommended in patients who failed to achieve biochemical hypoglycemia but developed manifestations due to hypoglycemia from the initial insulin administration.
